# Oxygenator Is the Main Responsible for Leukocyte Activation in Experimental Model of Extracorporeal Circulation: A Cautionary Tale

**DOI:** 10.1155/2015/484979

**Published:** 2015-05-04

**Authors:** Alessio Rungatscher, Maddalena Tessari, Chiara Stranieri, Erika Solani, Daniele Linardi, Elisabetta Milani, Alessio Montresor, Flavia Merigo, Beatrice Salvetti, Tiziano Menon, Giuseppe Faggian

**Affiliations:** ^1^Department of Surgery, Division of Cardiac Surgery, University of Verona, 37126 Verona, Italy; ^2^Section of General Pathology, Department of Pathology and Diagnostics, University of Verona, 37126 Verona, Italy; ^3^Department of Neurological and Movement Sciences, University of Verona, 37126 Verona, Italy

## Abstract

In order to assess mechanisms underlying inflammatory activation during extracorporeal circulation (ECC), several small animal models of ECC have been proposed recently. The majority of them are based on home-made, nonstandardized, and hardly reproducible oxygenators. The present study has generated fundamental information on the role of oxygenator of ECC in activating inflammatory signaling pathways on leukocytes, leading to systemic inflammatory response, and organ dysfunction. The present results suggest that experimental animal models of ECC used in translational research on inflammatory response should be based on standardized, reproducible oxygenators with clinical characteristics.

## 1. Introduction

Extracorporeal circulation (ECC) is essential for cardiopulmonary bypass (CPB) during conventional cardiac surgery and extracorporeal membrane oxygenation (ECMO) during extracorporeal life support [1].

Systemic inflammation after ECC is believed to contribute to various pathophysiological outcomes, including renal, pulmonary, and myocardial damage [2]. The underlying mechanism is likely to be multifactorial, involving operative trauma, hemodilution and endothelial damage by edema, ischemia/reperfusion of organs, and contact-activation of blood components during ECC [2, 3]. The latter is known to have a pivotal role even after recent improvements in materials and techniques. Despite these insights, the mechanisms that control systemic inflammatory response during ECC remain poorly characterized at the molecular level. To address this issue several experimental animal models have been described [4–10]. The rat model of ECC is more accessible and less expensive compared to large animal models. Indeed it allows large availability of assay also with transgenic, knock-out, and syngenic species. Most of the models currently described in literature are based on home-made oxygenators [4–10]. We have recently reported the validation of a rat model of ECC with a miniaturized hollow fiber oxygenator with current clinical standards produced by one of the leading companies in the clinical field [11].

In the present study we aimed to assess the role of oxygenator, manufactured with industrial standards and clinical characteristics, in the development of leukocyte activation and systemic inflammation in a rat model of ECC.

## 2. Methods

After institutional animal care committee approval, 20 male Wistar rats (400–450 g; Harlan, Udine, Italy) housed under standard laboratory conditions were anesthetized (sodium pentobarbital, 30 mg/kg intraperitoneally) and intubated through the oropharynx with a 14-gauge polyethylene tube. They were mechanically ventilated with a rodent respirator (Harvard Apparatus Inc., Holliston, Massachusetts). The tidal volume was 7 mL/kg and the respiratory rate was 50 to 60 breaths/min with an air-oxygen mixture (inspired oxygen fraction = 0.5). Ventilation was adjusted to keep an arterial carbon dioxide tension of 35 to 45 mmHg. Rats were secured supine on a heating board to maintain rectal temperature at 37°C during the surgical procedure before the initiation of ECC. The left femoral artery was cannulated with a heparinized 24-gauge Teflon catheter to monitor systemic arterial pressure and to collect arterial blood for gas analysis. Central cannulation was performed as previously described [11, 12]. In brief, after complete sternotomy, a venous cannula (a modified 4-hole 16-gauge Angiocath catheter) was advanced into the right atrium using a right transsuperior vena cava approach, allowing excellent drainage. The left common carotid artery was cannulated using an 18-gauge catheter advanced to the aortic arch. Another 18-gauge catheter was inserted into the right femoral vein and advanced into inferior vena cava.

Full heparinization (500 IU/kg) was assured after surgical preparation and immediately before ECC initiation to reduce overall blood loss. ECC was set up as previously described [11, 12]. The setup consisted of a venous reservoir, a roller pump, a hollow fiber oxygenator (Sorin, Mirandola, Italy), and a vacuum regulator with an applied pressure of −30 mm H_2_O to facilitate venous drainage, all connected by 1.6-mm internal diameter plastic tubing. Total priming volume was 10.5 mL, gas exchange surface was 450 cm^2^, and heat exchange surface was 15.8 cm^2^.

### 2.1. Study Design

Rats were randomized into two groups. The first group received the blood returned from the ECC circuit into the cannula positioned into the left common carotid artery.

In the second group of animals the oxygenator was not present in the ECC circuit and the blood was returned from the ECC circuit into the cannula allocated into the right femoral vein.

ECC was instituted in both groups at a flow rate of 120 mL/kg/min for 60 minutes. After the experiment, animals were humanely killed by anesthetic overdose.

### 2.2. Measurements

To access the inflammation induced by ECC in the two groups studied, blood samples were collected before and after ECC.

One hundred microliters of whole blood in ethylenediaminetetraacetic acid was determined by color immunofluorescent flow-cytometry analysis (FACSCalibur/BD CellQuest Pro software; Becton Dickinson, Tokyo, Japan).

Whole blood samples were incubated with Becton Dickinson (Franklin Lakes, NJ) lyse/fix buffer (containing phosphatase inhibitors) for 10 minutes at 37°C. Leukocytes were then isolated by centrifugation (300 g for 5 minutes) and washed once with phosphate-buffered saline. Cells were resuspended in 0.5 mL prechilled Becton Dickinson Perm Buffer III, vortexed, and incubated on ice for 30 minutes. After this, cells were washed twice with phosphate-buffered saline and resuspended in 0.5 mL Becton Dickinson StainBuffer (fetal bovine serum) before incubation for 30 minutes (at room temperature) with PE-Cy7- or PE-conjugated antibodies that recognized Ser529 phosphorylated RelA (p65; NF-*κ*B) or Thr180/Tyr182 phosphorylated p38 or isotype-matched controls, with subsequent washing and analysis by flow-cytometry (Becton Dickinson). Fluorescence of PE-conjugated p65 and PE-Cy7-conjugated p38 MAP kinase antibodies was quantified in granulocytes or mononuclear cells (identified by forward and side scatter plots) using Summit 4.3 software (Dako, Glostrup, Denmark).

The levels of TNF-*α* (Thermo scientific, Rockford, IL, USA), IL-6 (Thermo scientific, Rockford, IL, USA), and neutrophil elastase (NE, cloud-clone, Houston, TX, USA) were determined by Enzyme Linked Immunoadsorbent Assay (ELISA) kits, according to the manufacturer's instruction.

To access the inflammation in organ tissue, right lung was harvested immediately after euthanasia. Therefore it was fixed in 4% paraformaldehyde at 4°C overnight. Paraffin-embedded sections (3 *μ*m) were stained with hematoxylin and eosin (H&E) and examined by microscope analysis by a pathologist blinded to the experimental groups. The severity of lung injury was scored using a 5-point scale according to combined assessment of alveolar congestion, haemorrhage, accumulation of neutrophils in the airspace or vessel wall, and the thickness of alveolar wall/hyaline membrane formation with minimal 0 for normal lung histology and maximum 5 for most injured lung [13].

### 2.3. Statistic

Statistical analysis was performed using SPSS 11.0 software (SPSS Inc., USA). Results are reported as mean ± standard error (SE). Comparison between groups at a specific time point was determined by the Student *t* test. A *P* value <0.05 was considered significant.

## 3. Results

### 3.1. Hemodynamic Parameters

All the animals in both groups survived through the experiment.  Table 1 displays the hemodynamic and physiological parameters recorded before and after ECC. No differences between the two groups were demonstrated.

### 3.2. Inflammatory Signalling Pathways Activation on Leukocytes

**Figure 1:**
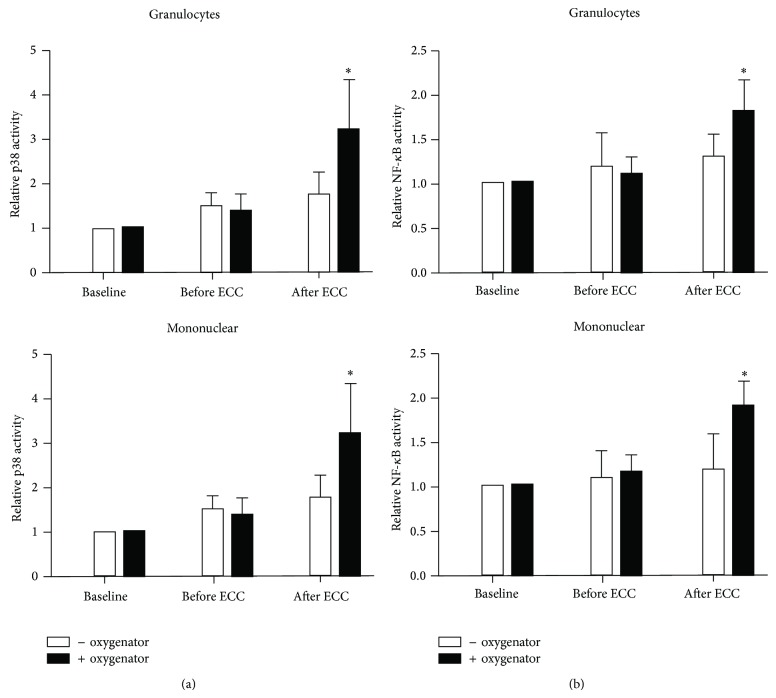
P38 and NF-*κ*B activation in leukocytes in response to ECC was significantly higher in presence of oxygenator. Peripheral blood samples were collected at baseline, before and after ECC. Leukocytes were fixed and permeabilized before intracellular staining using Alexa Fluor 568-conjugated antibodies that recognize (a) Thr180/Tyr182 phosphorylated p38 or (b) Ser529 phosphorylated NF-*κ*B or with isotype-matched antibodies as a control. After lysis of red blood cells, fluorescence of granulocytes or mononuclear cells was quantified by flow-cytometry (after gating of cells by size and granularity). Mean fluorescence levels were calculated after subtracting values from isotype-control antibodies. Mean values are shown with standard deviations. ^∗^ *P* < 0.05 between the two groups at the same time point.

The kinetics of leukocyte activation were determined by measuring p38 phosphorylation and NF-*κ*B phosphorylation in leukocytes. Intracellular staining and flow-cytometry demonstrated that ECC induced phosphorylation of both p38 (Figure 1(a)) and NF-*κ*B (Figure 1(b)) in mononuclear cells and granulocytes after ECC. Moreover the amount of leukocyte activation was significantly higher in ECC + oxygenator group.

**Figure 2:**
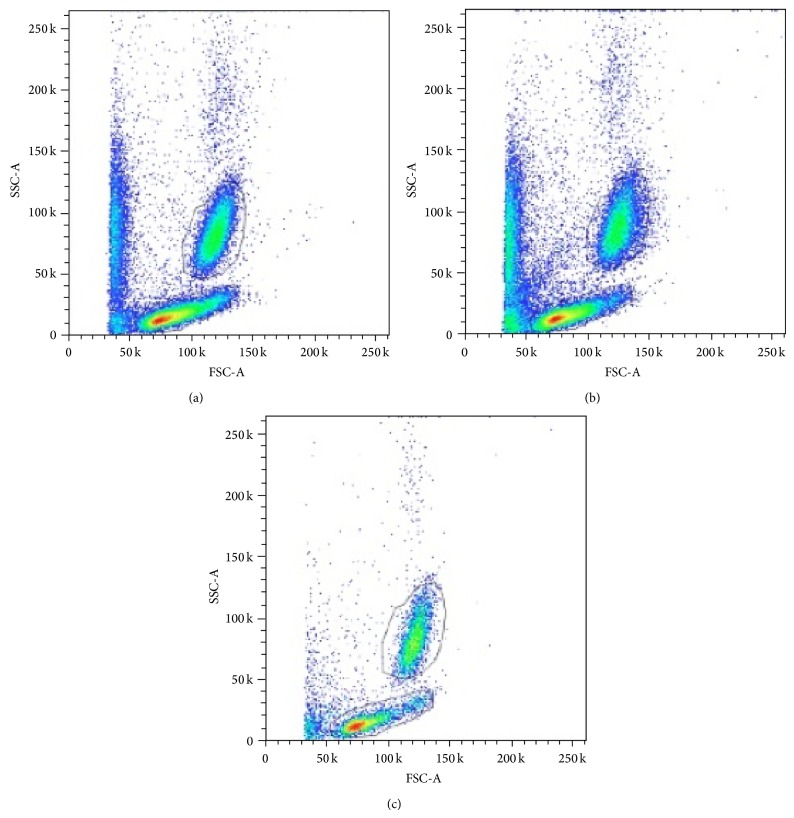
Representative flow-cytometry analysis at baseline (a), after ECC without oxygenator (b) and after ECC with oxygenator (c). A significant depletion of mononuclear cells and granulocytes after ECC + oxygenator is evident.

Figure 2 shows representative flow-cytometry analysis before ECC and after ECC in each group. A significant depletion of mononuclear cells and granulocytes after ECC + oxygenator is evident.

### 3.3. Inflammatory Response and Organ Injury

**Figure 3:**
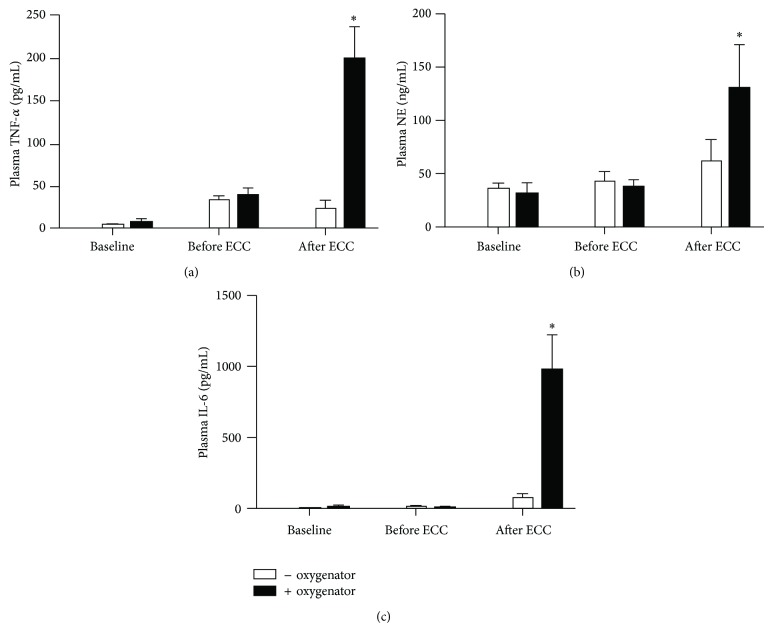
Systemic inflammatory response assessed by TNF-*α* (a), neutrophil elastase (NE) (b), and IL-6 (c) levels in plasma. ^∗^ *P* < 0.01 versus the other group at the same time point.

Plasma levels of TNF-*α* (Figure 3(a)), neutrophil elastase (NE) (Figure 3(b)), and IL-6 (Figure 3(c)) increased after ECC in both groups. However they were significantly higher in ECC + oxygenator compared to ECC − oxygenator (*P* < 0.01).

**Figure 4:**
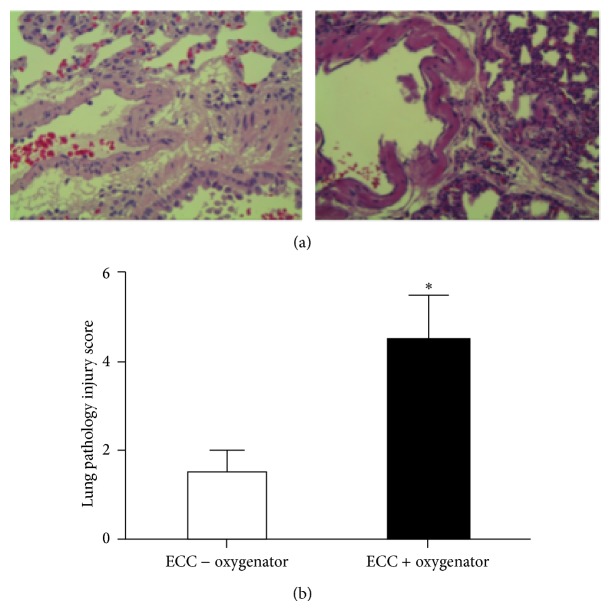
Lung injury. Representative histological images (magnification 40x) of right lung fixed in formaldehyde, sectioned, and stained with haematoxylin and eosin (a). The acute lung injury score was calculated (b). ^∗^ *P* < 0.01.

Rats receiving ECC + oxygenator demonstrated a recognized feature of pulmonary injury, characterized by edema, hemorrhage, increased thickness of alveolar wall, and inflammatory cells into alveolar spaces (Figure 4(a)). The lung injury scores were therefore significantly higher in ECC + oxygenator (4.50 ± 0.50) than in ECC − oxygenator (3.0 ± 1.0; *P* < 0.001) (Figure 4(b)).

## 4. Discussion

The present study has generated fundamental information on the role of oxygenator of ECC in activating inflammatory signalling pathways on leukocytes. By the use of a previously reported and validated rat model of ECC [11, 12], we have demonstrated for the first time that oxygenator has a pivotal role in triggering leukocyte activation and subsequent inflammatory response.

Given the recognized role of p38 and NF-*κ*B in the transcriptional induction of proinflammatory molecules [14–16], we have demonstrated that ECC + oxygenator leads to a significant phosphorylation (i.e., activation) of both p38 and NF-*κ*B in leukocytes. The underlying mechanism may involve contact-activation of blood components [17, 18]. Therefore it is possible to argue that the contact-activation between blood components and ECC surfaces is mainly produced in the oxygenator. The latter is indeed an essential component of ECC circuits in clinical setting [19]. Both CPB and ECMO are based on the performance of oxygenator to support circulatory and respiratory functions during cardiac surgery operations or critical cardiorespiratory diseases, respectively [20].

Surface coating of ECC circuits, oxygenator included, with heparin or phosphorylcholine has been proved to reduce the inflammatory response [21–24]. This is detectable not only on the basis of laboratory results but also by clinical parameters like reduced respiratory index and improved pulmonary and coagulation function in pediatric patients [21–23]. Therefore coated ECC equipment has become a clinical standard since beginning of 2000. However surface coating with either heparin or phosphorylcholine has not resolved the clinical problem of inflammatory response induced by ECC [24].

Many rat models of ECC have been proposed recently in order to assess molecular mechanisms responsible for activation of inflammatory response during ECC and to study possible therapeutic interventions [4–10]. All of these models are based on different home-made oxygenators. We have previously proposed a validated model of ECC in rat with a miniaturized hollow fiber oxygenator with clinical characteristics and manufactured with industrial standards by one of the leading producers of clinical oxygenators [11]. The present work demonstrated that a clinical relevant oxygenator has a major impact on leukocyte activation and systemic inflammatory response with implications in organ dysfunction. Therefore experimental studies aimed at investigating inflammatory response during ECC should be based on models with a standardized, reproducible, and clinical relevant oxygenator.

## 5. Conclusions

Oxygenator has a major role in leukocyte activation, inflammatory response, and organ dysfunction during ECC.

The present results suggest that experimental animal models of ECC used in translational research on inflammatory response should be based on standardized, reproducible oxygenators with clinical characteristics.

## Figures and Tables

**Table 1:** Hemodynamic and physiological parameters.

	Before ECC		After ECC	
	− oxygenator	+ oxygenator	− oxygenator	+ oxygenator
Heart rate (bpm)	287 ± 5	292 ± 8	314 ± 11	304 ± 10
MAP (mmHg)	58.3 ± 9.0	60.3 ± 8.3	50.5 ± 8.0	55.1 ± 9.7
Haematocrit (%)	34.5 ± 1.1	33.5 ± 1.8	11.2 ± 6.1	10.2 ± 5.8
Haemoglobin (g/dL)	12.8 ± 0.6	13.0 ± 0.9	4.8 ± 1.2	5.2 ± 0.9
WBC (×10^3^/mm^3^)	6.2 ± 1.8	5.5 ± 0.7	3.1 ± 0.4	2.7 ± 0.5
SaO_2_ (%)	98.2 ± 0.5	97.9 ± 0.4	95.2 ± 0.6	96.0 ± 1.1
PaO_2_ (mmHg)	72.3 ± 5.8	75.0 ± 4.1	69.0 ± 3.0	70.2 ± 9.0
PaCO_2_ (mmHg)	40.3 ± 5.9	43.0 ± 7.0	39.9 ± 4.0	42.3 ± 6.5
pH	7.32 ± 0.05	7.38 ± 0.04	7.22 ± 0.12	7.14 ± 0.20
BE (mmol/L)	−4.3 ± 1.2	−3.1 ± 1.5	−10.1 ± 4.7	−11.7 ± 6.2
HCO_3_ (mmol/L)	24.1 ± 7.8	22.0 ± 6.1	11.0 ± 10.1	16.1 ± 9.8

WBC = white blood cells; bpm = beats per minute; MAP = mean arterial pressure; SaO_2_ = arterial oxygen saturation; PaO_2_ = partial pressure of arterial oxygen; PaCO_2_ = partial pressure of arterial carbon dioxide; BE = base excess; HCO_3_ = standard bicarbonate. Data represent means ± SE.
